# Ethanol as a potential mosquito sample storage medium for RNA preservation

**DOI:** 10.12688/f1000research.20162.1

**Published:** 2019-08-14

**Authors:** Mirsha G. Torres, Allison M. Weakley, James D. Hibbert, Oscar D. Kirstein, Gregory C. Lanzaro, Yoosook Lee

**Affiliations:** 1 Vector Genetics Laboratory, Department of Pathology, Microbiology, and Immunology, University of California Davis School of Veterinary Medicine, Davis, CA, 95616, USA

**Keywords:** Sample storage, RNA preservation, field settings, mosquito, genetic analysis

## Abstract

Sample storage for downstream RNA analysis can be challenging in some field settings, especially where access to cryogenic materials or refrigeration/freezer facilities are limited. This has limited RNA-based studies on African malaria vectors collected in the field. We evaluated RNA quality after storing mosquito samples in three different sample preservation media over a 4-week period. Storing mosquito specimens in cold (4°C) media significantly improved yields of intact RNA. Our results indicate commercially available products perform well in keeping RNA integrity as advertised. Moreover, absolute ethanol may be an economical alternative for sample preservation that can be utilized in some resource-limited settings.

## Introduction

Samples to be used for downstream RNA analysis (e.g. RNA-Seq) are typically preserved by snap-freezing using liquid nitrogen or dry ice and then stored at -80°C until RNA extraction ^1, 2^. Several protocols have been published for preservation and extraction of genetic material from field collected samples ^3, 4^. Along with these protocols, there are products available to preserve nucleic acids from field collected specimens. These products include Allprotect Tissue Reagent (Qiagen, Hilden, Germany) and RNAlater (Thermo Fisher Scientific, Waltham, MA, USA). These reagents can stabilize tissue samples to maintain RNA content for one (RNALater) to six months (AllProtect) at mildly cold (4°C) temperatures. The duration can be increased to over one year if samples are stored in colder (-20°C) temperatures.

Optimal preservation of field collected samples to be used for gene expression studies require high quality nucleic acid, requiring preservation and stabilization of the RNA molecule ^5^. Unfortunately, the preservation of genetic material for expression studies based on field samples is difficult, and cryopreservation is often not possible. This is particularly applicable to field collections of *Anopheles* mosquitoes, which are the prime vector of malaria parasites ^6^ and exist in remote areas of Africa.

## Methods

### Mosquito samples

A total of 54 laboratory-reared *Anopheles coluzzii* mosquitoes from the UC Davis Vector Genetics Laboratory insectarium were subjected to various sample preservation conditions, as listed in Table 1. Three different sample preservation solutions were tested: Allprotect Tissue Reagent (Qiagen, Hilden, Germany), RNAlater (Thermo Fisher Scientific, Waltham, MA, USA), and 100% ethanol. Each set of samples was maintained in one of the three preservation solutions and subjected to two different temperature settings: typical refrigeration temperature (4°C) or at room temperature (28°C). A total of nine mosquito samples were stored in each of the six conditions listed in Table 1 for 4 weeks prior to RNA extraction.

**Table 1.** Sample size per and specimen storage conditions.

*Storage Medium*	*Storage Temperature (°C)*	N
AllProtect™	4	9
AllProtect™	28	9
RNALater™	4	9
RNALater™	28	9
Ethanol 100%	4	9
Ethanol 100%	28	9

### RNA extraction

Following a four-week sample preservation period, RNA was extracted from each mosquito sample using the Qiagen AllPrep DNA/RNA Mini Kit (Qiagen, Hilden, Germany) employing the manufacturer's protocol. The RNA concentration was measured using a Qubit RNA High Sensitivity kit and Qubit 2.0 instrument (Thermo Fisher Scientific, Waltham, MA, USA) using the manufacturer’s protocol. The RNA fragment size distribution was examined using the Agilent High Sensitivity RNA Analysis kit and TapeStation 4200 instrument (Agilent, Santa Clara, CA, USA), and the dominant peak size and proportion of long (>1000 bp) fragments were recorded. Typical RNA integrity number (RIN) which measures the 28S and 18S rRNA ratio was not used due to negligible 28S peaks, which is typical for insect RNA extracts ^7^.

### Data analysis

Mann-Whitney tests were conducted using the scipy module version 1.2.0 ^8^ in the Jupyter notebook ^9^ version 4.1. environment. Plots were generated using Matplotlib version 3.1.0 ^10^.

## Results and Discussion

**Figure 1.**
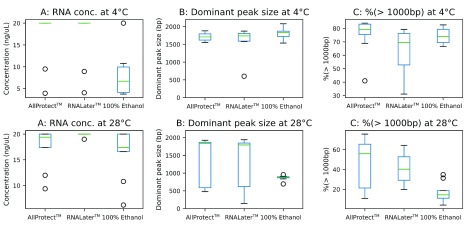
Comparison of sample preservation media at two different temperature settings.

Results for each sample are available as *Underlying data* ^11^. Preservation conditions that resulted in the highest concentration of longer RNA fragments (>1000 bp) were considered to be best for downstream genetic analysis, as opposed to those resulting in degraded RNA (fragment size < 1000 bp). As expected, storage at 4°C generally preserved RNA integrity better than 28°C. There were no significant differences in total RNA concentration or dominant peak size between samples stored in AllProtect™ or RNALater™ at either storage temperature (Mann-Whitney test, P>0.05, Figure 1). Samples stored in absolute ethanol, however, showed a significant increase in RNA yield (Mann Whitney Test, P=0.0065) and significant decrease in dominant peak size (Mann-Whitney test, P=0.00020) when stored at 28°C. We observed a significant reduction in long fragment (>1000 bp) RNA in samples stored at 28°C than at 4°C regardless of the preservation solution (Mann-Whitney test, ɑ < 0.05). These results suggest, as expected, that higher temperatures accelerates tRNA degradation. Degradation was greater in absolute ethanol, decreasing the proportion of long (>1000bp) fragments from 74.1% (±5.6 STD) at 4°C to 16.9% (±10.1 STD) at 28°C. RNAlater and AllProtect™ maintained a >40% content of RNA fragments of ≥1000 bp.

At 4°C no significant difference was observed in dominant RNA peak size (1600-1814 bp) and proportion of long (>1000bp) RNA fragments (62.2–74.9%) among the three preservation media tested (Mann-Whitney test, P>0.05). The only significant difference was a lower concentration of RNA in ethanol compared with the other preservation solutions (Mann-Whitney test, P<0.0081).

Absolute ethanol did not preserve RNA integrity at 28°C, with only 16.9% (±10.1 STD) of RNA content composed of 1000 bp or longer fragments. However, the peak RNA fragment size for samples stored in absolute ethanol at 28°C was 869 bp (±73 STD) showing little variation ( Figure 1) yet similar concentrations as the other two preservation solutions. This quality may be sufficient to conduct downstream RNA analysis for real-time PCR or RNA-Seq.

Overall, samples stored in RNAlater™ or AllProtect™ at 4°C provide satisfactory preservation of RNA content from field collected samples after 4 weeks in storage. Absolute ethanol may provide an economical alternative in resource-constrained field settings. Currently in the USA, AllProtect™ is available at ~$6.5/mL, RNAlater™ at $0.9-1.4/mL, and 200 proof lab grade ethanol at $0.1-0.6/mL. When stored at 4°C, absolute ethanol may be a viable alternative to commercially available products. Although RNA stored in ethanol at 28°C will degrade faster, it nonetheless maintained fragment sizes over 800 bp after 4 weeks in storage. Future evaluation of RNA quality utilizing real time PCR or RNA-seq may be necessary to elucidate whether ethanol is indeed an adequate sample preservation solution for RNA preservation. For practical applications, keeping specimens in a commercial RNA storage solution at 4°C maximizes maintenance of RNA integrity.

## Data availability

### Underlying data

Open Science Framework: Sample storage condition testing for RNA preservation. https://doi.org/10.17605/OSF.IO/BRNPV ^11^.

This project contains data on RNA source, storage, concentration, dominant peak size and quality from each sample assessed in this study.

Data are available under the terms of the Creative Commons Attribution 4.0 International license (CC-BY 4.0).
