# Supplementary material for: Ethanol as a potential mosquito sample storage medium for RNA preservation
Source: F1000Res. 2019 Aug 14;8:1431. [Version 1] doi: 10.12688/f1000research.20162.1 (PMC6713064; doi:10.12688/f1000research.20162.1)
Supplement: Peer review history

# Peer review: Reviewer response for version 1

**DOI:** 10.5256/f1000research.22147.r52465

**Published:** 2019-08-27

**Authors:** Megan A. Riddin

**Affiliations:** 1 UP Institute for Sustainable Malaria Control (UP ISMC), Faculty of Health Sciences, University of Pretoria, Pretoria, South Africa

This article addresses the efficacy of commercially available RNA preservation media and absolute ethanol at two temperatures, room temperature (28°C) and cold (4°C). The article focuses upon a critical topic that is the success of preservation of samples for downstream RNA analysis, particularly to find a reliable source in limited field source settings. The outcome of this study has direct application to a number of fields, including the study of malaria vectors which are often collected in preservation limited settings with a complete lack of freezer facilities for long periods.

 This paper is a significant addition to the literature and has been addressed well. The selection of only a 4 week testing period is short, however, I feel the outcomes of this study can be immediately applied in a number of research fields, particularly research on mosquito disease vectors, as most field collections do not extend past such a period. By including absolute ethanol and a two RNA-preservative media options, this enables the covering of a cheaper and readily available option as well as other more stable, but more expensive and may be difficult to obtain, options for regions where snap-freezing is not an option.

**More specific comments:**

 The authors covered the problem, objective and study highlights well in the abstract and state that absolute ethanol is a viable option in resource-limited settings.

 I can highly appreciate that this was a direct study for the preservation of malaria vectors in the field which is predominantly in resource limited regions. It would complement the methods to include reasoning on selection of insectary-reared *Anopheles coluzzi*i, 9 individuals and the 4 week period. This would be of interest to researchers who would replicate the methodology or adapt it for their sample and preservations needs. I commend the authors on including RNA concentration as well as fragment size distribution analysis, offering a comprehensive effect of the preservation method on RNA integrity.

 The results of the study are clearly and concisely described, and significance provided. The most important results are communicated and basis for conclusions are of high standard. The figures are understandable and visually descriptive. It is excellent that the authors further include an expense comparison for the appreciation of the use of absolute ethanol in funding and resource limited studies, as well as highlight the need for further evaluation of the preservation methods for real time PCR and RNA-seq.

 Overall this is a worthy article which is a good addition to literature, and has direct application for RNA preservation.

I confirm that I have read this submission and believe that I have an appropriate level of expertise to confirm that it is of an acceptable scientific standard.

# Peer review: Reviewer response for version 1

**DOI:** 10.5256/f1000research.22147.r52797

**Published:** 2019-08-27

**Authors:** Tara Roth

**Affiliations:** 1 San Mateo County Mosquito and Vector Control District, Burlingame, CA, USA

This is a useful methods paper to help clarify any misconceptions researchers might have about what is strictly necessary in order to preserve genomic specimens. I feel like this information could be highly useful for field researchers but also as information to help students just starting research projects.

 Overall, I don't have many comments to make. The sample size is somewhat small but appears statistically valid. The one thing that would have been really nice to see is a comparison of the study findings to RNA degradation under ideal conditions (-80) as well as with no preservation methods - just dry on the table.

I confirm that I have read this submission and believe that I have an appropriate level of expertise to confirm that it is of an acceptable scientific standard.
